# Background parenchymal enhancement in ultrafast MRI: Inter-reader agreement before and after training, and correlations with prognostic factors of breast cancer

**DOI:** 10.1016/j.ejro.2026.100820

**Published:** 2026-10-14

**Authors:** Ken Yamaguchi, Kanto Ichinohe, Takahiko Nakazono, Ryoko Egashira, Mizuki Iyadomi, Takeshi Imaizumi, Katsuya Maruyama, Dominik Nickel, Osamu Togao

**Affiliations:** a Department of Radiology, Faculty of Medicine, Saga University, 5-1-1 Nabeshima, Saga City, Saga, 849-8501, Japan; b Department of Radiology, Kurume University School of Medicine, 67 Asahi-machi, Kurume, Fukuoka, 830-0011, Japan; c Department of Radiology, Saga-ken Medical Centre Koseikan, 400 Nakabaru, Kasemachi, Saga City, Saga, 840-8571, Japan; d Satsuma Lab, Hawkes Institute, University College London, 90 High Holborn, London, WC1V6BH, UK; e MR Research & Collaboration Department, Siemens Healthcare K.K., Gate City Osaki West Tower, 1-11-1 Osaki, Shinagawa-ku, Tokyo, 141-8644, Japan; f MR Research & Clinical Translation, Siemens Healthineers AG, Allee am Roethelheimpark 2, Erlangen, 91052, Germany

**Keywords:** Breast, Magnetic resonance imaging, Ultrafast, Background parenchymal enhancement, Inter-reader agreement

## Abstract

**Purpose:**

To assess the inter-reader agreement in background parenchymal enhancement (BPE) categorization on ultrafast dynamic contrast-enhanced (DCE) MRI before and after training and its relationship with breast cancer prognostic factors.

**Materials and methods:**

We retrospectively analyzed the cases of 220 patients (median, age 60 years; 220 breast cancers) who underwent ultrafast DCE MRI. BPE was qualitatively classified as minimal, mild, moderate, or marked by three readers: Reader1 (R1), a radiology resident in the second year of training; Reader2 (R2), an experienced general radiologist with 28 years of experience in diagnostic radiology; and Reader3 (R3), an experienced breast radiologist with 25 years of experience in breast MRI. Inter-reader agreement was evaluated using weighted kappa (κ) values and was reassessed between R1 and R3 after intensive training. Associations between BPE levels and prognostic factors were also evaluated.

**Results:**

Inter-reader agreement was substantial with weighted κ values of 0.679–0.742 for R1 versus R2, 0.612–0.665 for R1 versus R3, and 0.626–0.689 for R2 versus R3. Agreement between R1 and R3 improved slightly after training but remained substantial. No significant associations were observed between visual BPE classification and prognostic factors, including hormone receptor status, HER2 status, and proliferation markers.

**Conclusion:**

Inter-reader agreement for BPE assessment on ultrafast DCE MRI was substantial and improved slightly after training. No significant relationship was observed between ultrafast BPE and breast cancer prognostic factors.

## Highlights

• The inter-reader agreement for BPE in ultrafast DCE MRI was substantial.
• The inter-reader agreement showed slight improvement after training.
• Visual BPE classification was not associated with breast cancer prognostic factors.

## 1 Introduction

Breast magnetic resonance imaging (MRI) has the highest sensitivity for breast cancer detection [1] and is used for various purposes in breast imaging [2]. In breast MRI examinations, background parenchymal enhancement (BPE) can interfere with the diagnosis [3]. The term 'BPE' refers to the enhancement of normal breast tissue observed on breast MRI [3], [4]. BPE is visually classified into four qualitative categories: minimal, mild, moderate, and marked [4]. On conventional dynamic contrast-enhanced (DCE) MRI, BPE is typically assessed approximately 90 s after contrast material administration [4]. Strong BPE can obscure lesions and thereby make lesion detection difficult. The degree of BPE is known to depend on several factors, including age, menopausal status, menstrual cycle, and the use of medications such as aromatase inhibitors [3], [5], [6].

In conventional DCE MRI, reported inter-reader agreement for BPE has varied considerably, with kappa (κ) values ranging from 0.24 to 0.9 [7], [8], [9]. In one study, inter-reader agreement relatively low even after intensive training in BPE classification [7].

Ultrafast DCE MRI, which enables image acquisition with substantially higher temporal resolution than conventional DCE MRI, has recently gained attention [10], [11], [12], [13]. On ultrafast DCE MRI, lesions typically enhance before BPE becomes prominent, potentially facilitating the lesion detection in cases with strong BPE [14]. However, substantial BPE may still be present on ultrafast MRI and may affect detection and morphological assessment of breast lesions, although the morphological evaluation of lesions has been attempted using ultrafast MRI [15], [16].

Our review of the relevant literature identified only a few studies that specifically examined inter-reader agreement for BPE on ultrafast DCE MRI; these studies reported substantial to almost perfect agreement (κ values, approximately 0.7–0.9) [9], [14]. BPE has also been investigated as a potential risk and prognostic factor for breast cancer, and several studies have examined its relationships with overall or recurrence-free survival [17], [18], [19]. Although associations between breast lesions and various prognostic factors (e.g., hormone receptor status, HER2 status, proliferative markers, and other) have been studied [20], [21], [22], [23], [24], [25], [26], we have found no published study examining the relationship between visually assessed normal breast BPE on ultrafast MRI and breast cancer prognostic factors. We therefore conducted the present study to assess (*i*) the inter-reader agreement in BPE categorization before and after training and (*ii*) the relationships between BPE on ultrafast DCE MRI and prognostic factors of breast cancer.

## 2 Materials and methods

### 2.1 Patients

This retrospective study was approved by our institutional review board (protocol no. 2017–09–06), and the requirement for patients' written informed consent was waived. We reviewed 241 patients with 247 cancers who underwent breast MRI at our institution between August 2018 and February 2022. We excluded patients with bilateral cancers, a history of ipsilateral breast cancer surgery, technical errors in image acquisition, or missing ultrafast data.

### 2.2 MRI technique

All DCE MRI examinations were performed using a 3 T MR system (MAGNETOM Prisma Fit; Siemens Healthineers, Erlangen, Germany) with a dedicated 18-channel breast coil. Ultrafast imaging was acquired using a volume interpolated breath-hold examination (VIBE) research sequence with compressed sensing (CS) reconstruction. The scan protocol consisted of one precontrast phase and 30 postcontrast phases (the second phase started simultaneously with contrast injection), with a temporal resolution of 2.9 s per phase. The total postcontrast scan time was 87 s. Sequence parameters were as follows: TR/TE, 3/1.2 ms; flip angle, 12°; voxel size, 0.9 × 0.9 × 2.5 mm. Gadobutrol (Gadovist, Bayer Schering Pharma, Berlin, Germany) was administered intravenously at 0.1 mmol/kg, followed by a 20-mL saline flush at a rate of 2.5 mL/sec.

Before the ultrafast DCE sequence, axial diffusion-weighted images based on readout-segmented echo-planar imaging (EPI), axial gradient-echo T1-weighted images, fat-suppressed axial spin-echo T2-weighted images, and one pre-contrast fat-suppressed axial T1-weighted image using a VIBE sequence for the conventional DCE study were obtained. After completion of the ultrafast DCE sequence, two contrast-enhanced fat-suppressed axial T1-weighted images using VIBE sequences for the conventional DCE study and one contrast-enhanced fat-suppressed sagittal T1-weighted images using VIBE sequences as high-spatial-resolution imaging were obtained. The postcontrast axial T1-weighted acquisition began immediately after completion of the ultrafast DCE sequence, and the second began 270 s after the contrast material injection. High-resolution sagittal T1-weighted images were acquired between the first and second post-contrast DCE acquisitions. Further details of the breast MRI protocol have been described previously [26], [27].

### 2.3 Image analyses

Each case's BPE was classified into one of four categories (minimal, mild, moderate, and marked) defined by the American College of Radiology (ACR) Breast Imaging-Reporting and Data System (BI-RADS) MRI lexicon [4]. BPE was evaluated on maximum intensity projection (MIP) images of the breast contralateral to the cancer at phases 10, 20, and 30 (corresponding to 29, 58, and 87 s after the contrast material injection, respectively). Images from the BI-RADS atlas were used as references. Three readers independently evaluated the BPE levels:

• Reader 1: a radiology resident in the second year of training
• Reader 2: an experienced general radiologist with 28 years of experience in diagnostic radiology
• Reader 3: an experienced breast radiologist with 25 years of experience in breast MRI

Readers 1 and 2 were blinded to histopathological prognostic factors, surgical outcomes, and patient demographic information including age and menopausal status, during BPE assessment. Reader 3 was blinded to histopathological prognostic factors and surgical outcomes; however, because Reader 3 was a breast radiologist involved in routine clinical interpretation at our institution, complete blinding to age and menopausal status could not be guaranteed. To assess the effects of training on BPE classification, Readers 1 and 3 reviewed typical cases together over a six-months period after the initial reading. BPE levels were re-evaluated one month after completion of this training period. We then calculated the inter-reader agreement between Readers 1 and 3 and compared the post-training results with the pre-training results.

We also investigated whether the BPE levels were associated with various prognostic markers in the invasive cancer cases (n = 187) at phases 20 and 30 of ultrafast DCE MRI. For this analysis, we determined an "integrated BPE" score as follows: when Readers 1 and 2 agreed on the BPE classification, that classification was used; when they disagreed, the classification assigned by the experienced breast radiologist (Reader 3) was used. Integrated BPE values were dichotomized into "Low BPE" (minimal or mild) and "High BPE" (moderate or marked) for the statistical analyses.

### 2.4 Statistical analyses

Weighted κ values were used to assess agreement between pairs of readers. For Association between BPE and prognostic factors were evaluated using the Wilcoxon rank-sum test, χ^2^- test, or Fisher's exact test, as appropriate. A p value < 0.05 was considered statistically significant. Statistical analyses were performed using IBM SPSS Statistics (IBM, Chicago, IL) and JMP statistics ver. 16.1.0 (SAS Institute Japan, Tokyo, Japan).

## 3 Results

The final study population consisted of 220 patients (median age, 60 years) with 220 cancers. The patient selection flowchart is shown in Fig. 1.

**Fig. 1:**
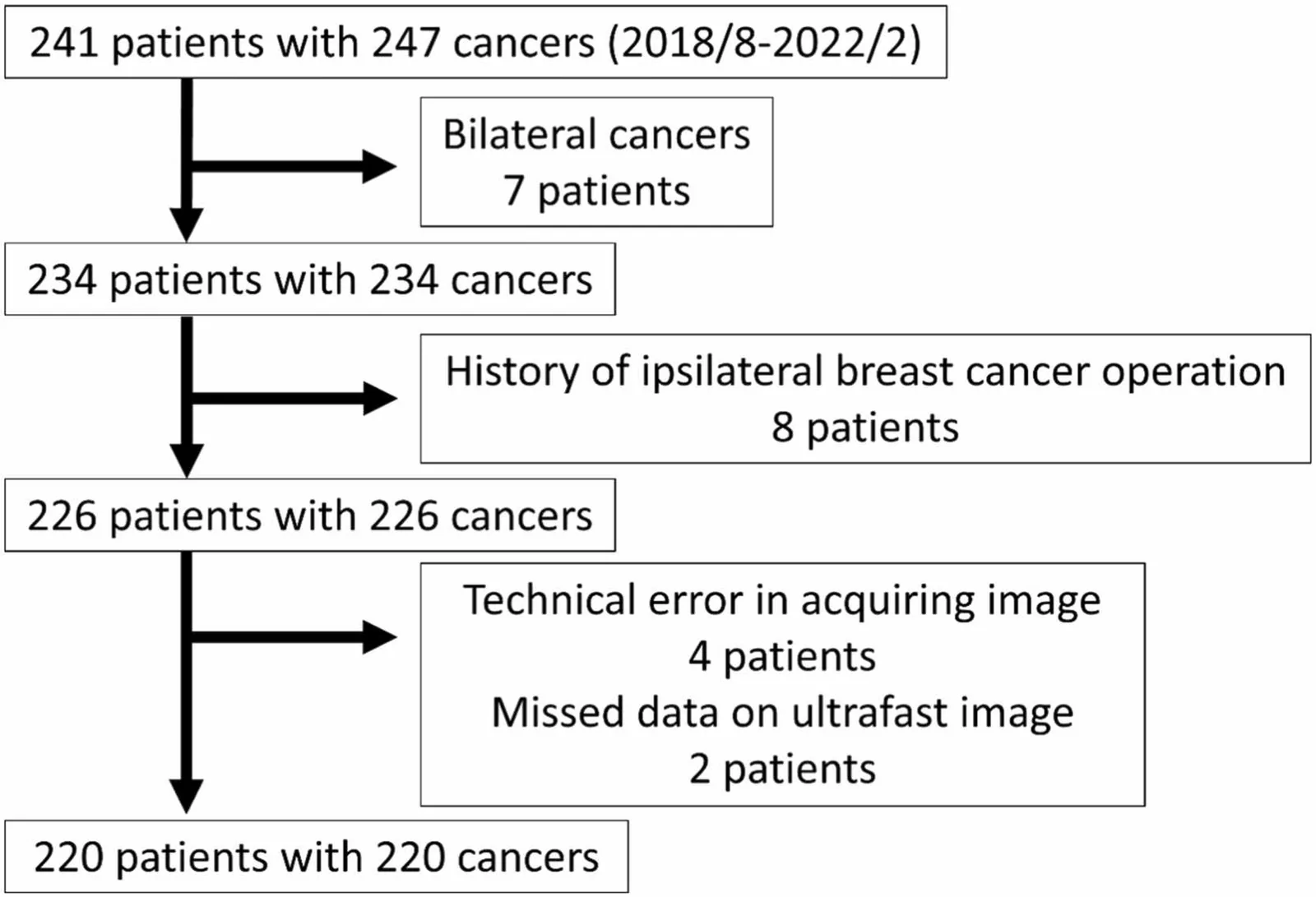
Flow chart of the patient selection.

### 3.1 Inter-reader agreement

Weighted κ values for inter-reader agreement on ultrafast DCE MRI ranged from 0.612 to 0.742, indicating substantial agreement (Table 1). Agreement between radiology resident (Reader 1) and the general radiologist (Reader 2) tended to be higher than the agreement between either of these readers and the breast radiologist (Reader 3). Readers 1 and 2 tended to assign higher BPE scores than Reader 3 (Table 2, Fig. 2).

**Table 1:** Inter-reader agreement of BPE shown by ultrafast dynamic contrast-enhanced (DCE) MRI before the BPE training.

	Weighted κ-value		
	Phase 10	Phase 20	Phase 30
Reader 1 vs. Reader 2	0.679	0.729	0.742
Reader 1 vs. Reader 3	0.661	0.665	0.612
Reader 2 vs. Reader 3	0.626	0.689	0.662

Reader 1: Radiology resident in the second year of training. Reader 2: Experienced general radiologist with 28 years of experience in diagnostic radiology. Reader 3: Experienced breast radiologist with 25 years of experience in breast MRI.

**Table 2:** The results of the three readers' BPE evaluations before the training.

		**Minimal**	**Mild**	**Moderate**	**Marked**
Phase 10	Reader 1	165	39	12	4
	Reader 2	161	35	21	3
	Reader 3	188	21	8	3
Phase 20	Reader 1	123	65	25	7
	Reader 2	143	41	29	7
	Reader 3	163	35	16	6
Phase 30	Reader 1	114	72	27	7
	Reader 2	134	49	30	7
	Reader 3	163	35	16	6

Reader 1: Radiology resident in the second year of training. Reader 2: Experienced general radiologist with 28 years of experience in diagnostic radiology. Reader 3: Experienced breast radiologist with 25 years of experience in breast MRI.

**Fig. 2:**
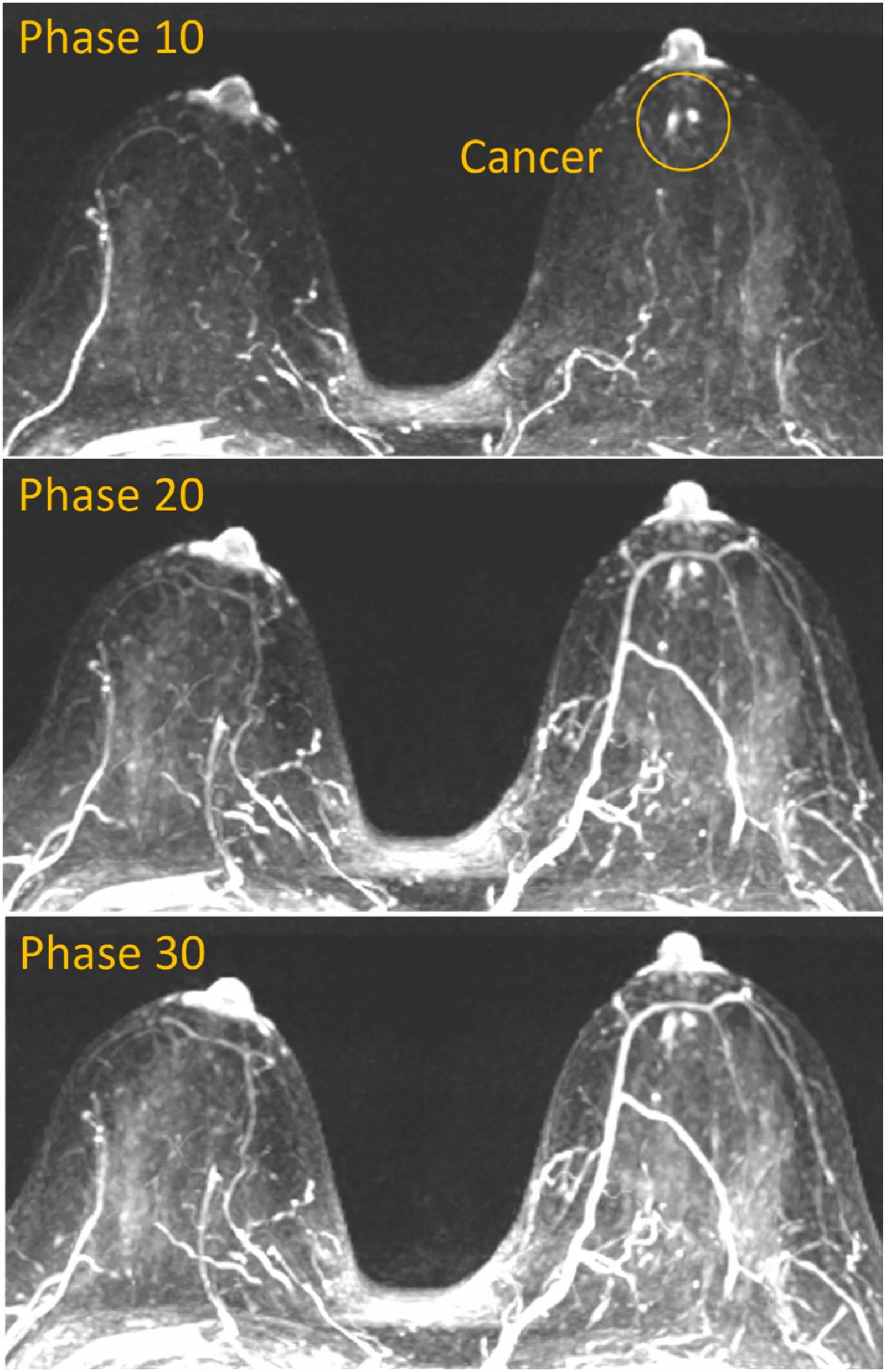
A 37-year-old woman with ductal carcinoma in situ of the left breast. At phase 10, Readers 1 and 3 scored the patient's background parenchymal enhancement (BPE) as 'minimal,' but Reader 2 scored it as 'mild.' At phases 20 and 30, the scores given by Readers 1 and 2 were 'moderate'; Reader 3's score was 'minimal.' Agreement by all three readers was not achieved at any of the phases. At phases 10 and 20, Readers 1 and 2 may have overestimated the BPE due to the increased visibility of vascular structures. Reader 1: a senior resident, Reader 2: an experienced general radiologist, Reader 3: an experienced breast radiologist.

### 3.2 Effects of training

Comparison of BPE classifications by Readers 1 and 3 before and after BPE classification training showed slight improvement in weighted κ values, although agreement remained within the substantial range (Table 3): phase 10, from 0.661 to 0.700; phase 20, from 0.665 to 0.721; and phase 30, from 0.612 to 0.695.

**Table 3:** The inter-reader agreement regarding the classification of BPE based on ultrafast DCE MRI before and after the training.

**Reader 1 vs. Reader 3**	**Weighted κ-value**		
	**Phase 10**	**Phase 20**	**Phase 30**
Before training	0.661	0.665	0.612
After training	0.7	0.721	0.695

Reader 1: Radiology resident in the second year of training.

Reader 3: Experienced breast radiologist with 25 years of experience in breast MRI.

After the training, Reader 1 tended to assign lower scores, correcting a previous tendency to interpret vessels, lesions, or artifacts as BPE.

### 3.3 Correlations between BPE and prognostic factors

As shown in Table 4, the analysis of the integrated BPE scores at phases 20 and 30 revealed no significant differences between the Low BPE and High BPE groups in tumor size, estrogen receptor (ER) status, progesterone receptor (PgR) status, HER2 status, Ki-67 index, nuclear grade, or lymph node metastasis.

**Table 4:** The correlations between the integrated BPE groups and the prognostic factors for breast cancer.

		**Phase 20**			**Phase 30**		
		**Higher BPE**	**Lower BPE**	**p-value**	**Higher BPE**	**Lower BPE**	**p-value**
Size, mm*		21	21	0.8793	21	21	0.8359
ER	Positive	19	143	0.7447	21	141	07444
	Negative	2	23		2	23	
PgR	Positive	19	129	0.2557	21	127	0.1722
	Negative	2	37		2	37	
HER2	Positive	1	21	0.4765	1	21	0.3208
	Negative	20	145		22	143	
Ki-67*		10%	10%	0.333	10%	10%	0.1659
Nuclear grade	1	15	112	0.5533	16	111	0.3631
	2	4	23		5	22	
	3	2	29		2	29	
LN metastasis	Positive	4	32	0.7941	5	26	1
	Negative	25	158		26	157	

* Values are medians. Higher BPE group: moderate, marked. Lower BPE group: minimal, mild. ER: estrogen receptor,

## 4 Discussion

In the present study, inter-reader agreement for visual BPE classification on ultrafast DCE MRI was substantial, with weighted κ values ranging from 0.612 to 0.742. Previous studies specifically evaluating BPE on ultrafast DCE MRI reported higher agreement, with κ values of approximately 0.7–0.9 [9], [14]. The slightly lower agreement in our study may reflect differences in reader composition. Previous studies appear to have involved readers with experience in breast imaging. For example, Bode et al. included two breast radiologists with 10 and 5 years of experience in breast MRI, respectively, both of whom also had 2 years of experience interpreting ultrafast MRI [9]. whereas detailed information on reader experience was not provided by Honda et al [14]. In contrast, our study included readers with markedly different levels of radiological and breast imaging experience, ranging from a second-year radiology resident to a general radiologist with 28 years of diagnostic radiology experience, and a breast radiologist with 25 years of breast MRI experience. Thus, our results extend the previous observations by showing that substantial agreement can be achieved even across readers with heterogeneous breast imaging experience, a setting that may more closely reflect routine clinical practice.

The pattern of disagreement also provides context for the difference from previous reports. Reader 1 and 2 tended to assign higher BPE categories than Reader 3, particularly when vascular structures became more conspicuous (Table 2, Fig. 2). This suggests that reader experience specific to breast MRI may influence the distinction between the true parenchymal enhancement and other structures (e.g., vessels) or artifacts. This interpretation is consistent with the slight improvement observed after training, when Reader 1 tended to assign lower BPE scores and weighted κ values between Readers 1 and 3 increased at all three evaluated phases. These findings suggest that targeted training focused on distinguishing BPE from other structures (e.g., vessels) and artifacts may improve consistency, particularly for readers with limited breast MRI experience.

Our findings can also be considered in relation to conventional DCE MRI. Previous studies of conventional DCE MRI reported a broad range of inter-reader agreement for BPE (κ values: 0.24–0.9) [7], [8], [9]. In addition, Melsaether et al. reported only modest improvement after training, with increasing from 0.36 to 0.48 [7].

In contrast, agreement our study was already substantial before training and remained substantial after training with κ values between Reader 1 and 3 increasing from 0.612–0.665 to 0.695–0.721. One possible explanation is that earlier assessment on ultrafast DCE MRI may limit the extent of BPE, resulting in a narrower distribution across the four BPE categories (minimal, mild, moderate, and marked) than that observed on later postcontrast images. Although this improvement was slight, there was still substantial agreement. Further improvements in inter-reader agreement may require consistent reading and discussion by all readers within the group.

Regarding the clinical significance of BPE, we observed no significant association between the ultrafast BPE classifications and breast cancer prognostic factors. Several studies using conventional DCE MRI have reported associations between BPE and breast cancer survival outcomes [17], [18], [19]. We did not investigate the relationship between BPE and breast cancer survival outcomes because many of the present cases were examined within a short follow-up period after surgery. The above-mentioned studies used segmented quantitative data rather than the visual evaluation we used herein [17], [18], [19]. In addition, the number of cases in the present study was relatively small (n = 220). Moreover, BPE values evaluated with the use of ultrafast DCE MRI are acquired early after contrast enhancement, resulting in less distribution variation compared to the BPE evaluated with conventional DCE MRI. Ultrafast DCE MRI may therefore not be suitable for evaluating the relationship between BPE and prognoses. Alternatively, a quantitative BPE evaluation might be needed instead of a four-point visual assessment using ultrafast DCE MRI. Further studies with larger numbers of cases are necessary.

Our study has some limitations to consider. First, it was a single-institution retrospective study with a relatively small number of cases. Second, menstrual cycles a known factor affecting BPE were not controlled at the time of imaging. Third, although all readers were blinded to histopathological prognostic factors and surgical outcomes, complete blinding of Reader 3 to age and menopausal status could not be guaranteed because this reader was involved in routine clinical breast MRI interpretation at our institution. This may have introduced expectation bias into the visual assessment of BPE. Future multicenter studies with larger cohorts and longer follow-up periods are warranted to fully elucidate the clinical value of BPE assessment on ultrafast DCE MRI.

## 5 Conclusion

Inter-reader agreement for background parenchymal enhancement (BPE) assessment on ultrafast DCE MRI was substantial and improved slightly after training. No significant relationship between ultrafast BPE levels and breast cancer prognostic factors was identified in this study.

## CRediT authorship contribution statement

**Ken Yamaguchi:** Writing – original draft, Software, Methodology, Formal analysis, Conceptualization. **Kanto Ichinohe:** Formal analysis. **Takahiko Nakazono:** Writing – review & editing, Formal analysis. **Ryoko Egashira:** Writing – review & editing. **Mizuki Iyadomi:** Writing – review & editing. **Takeshi Imaizumi:** Formal analysis. **Katsuya Maruyama:** Writing – review & editing. **Dominik Nickel:** Writing – review & editing. **Osamu Togao:** Supervision.

## Ethics declaration

The authors have NOT obtained informed consent from participants or their legal representatives. The requirement for written informed consent was waived by the Institutional Review Board because of the retrospective nature of the study and the use of existing clinical data.

This study was performed in compliance with relevant laws, regulatory frameworks and guidelines where the research took place. This study was approved by the Saga University Hospital (Approval No. 2017–09–06).

## Declaration of Generative AI and AI-assisted technologies in the manuscript preparation process

During the revision of this manuscript, the authors used ChatGPT (OpenAI) to improve the language, clarity, readability, and organization of the text. After using this tool, the authors reviewed and edited the content as needed and take full responsibility for the content of the publication.

## Declaration of Competing Interest

K. Maruyama is an employee of Siemens Healthcare K.K. D. Nickel is an employee of Siemens Healthineers AG. The other authors declare no conflicts of interest.

## Data Availability

The datasets generated and/or analyzed during the current study are available from the corresponding author on reasonable request.
